# Regioselective DNA Modification and Directed Self-Assembly of Triangular Gold Nanoplates

**DOI:** 10.3390/nano9040581

**Published:** 2019-04-09

**Authors:** Guoqing Wang, Yao Zhang, Xingguo Liang, Tohru Takarada, Mizuo Maeda

**Affiliations:** 1 Bioengineering Laboratory, RIKEN Cluster for Pioneering Research, 2-1 Hirosawa, Wako, Saitama 351-0198, Japan; mizuo@riken.jp; 2 College of Food Science and Engineering, Ocean University of China, 5 Yushan Road, Qingdao 266003, China; yzhangcncn@163.com (Y.Z.); liangxg@ouc.edu.cn (X.L.); 3 Laboratory for Marine Drugs and Bioproducts of Qingdao National Laboratory for Marine Science and Technology, Qingdao 266237, China

**Keywords:** DNA, gold, nanoplate, nanotriangle, regioselective modification, self-assembly

## Abstract

As a class of emerging nanoparticles, gold nanotriangles (AuNTs) are characterized by unique structural anisotropy and plasmonic properties. The organization of AuNTs into well-defined architecture potentially promises collective properties that are difficult to produce by individual AuNTs. To date, however, the orientation-controlled self-assembly of AuNTs has been achieved with limited success. Here, we describe an effective and straightforward approach to induce directed self-assembly of AuNTs. By taking advantage of the uneven chemical reactivity of AuNT surfaces, we implement regioselective modification of the edges and the top/bottom surfaces with two different double-stranded DNA (dsDNA) sequences. By means of terminal single base pairing/unpairing, controlled assembly of the dsDNA-modified AuNTs evolves in a face-to-face or edge-to-edge manner based on blunt-end stacking interaction on an intentional region of the AuNTs, along with entropic repulsion by unpaired terminal nucleobases on the other region. This approach could be useful for achieving directed self-assembly of other anisotropic nanoparticles.

## 1. Introduction

Over the past decades, gold nanoparticles (AuNPs) featuring unique physicochemical properties have found broad application, ranging from biosensing [1] and therapeutics [2,3] to photocatalysis [4,5]. Of the many shaped nanomaterials [6], gold nanoplates represent an emerging class of anisotropic nanostructure with a lateral dimension larger than their thickness [7,8,9]. Recent research advances have witnessed the increasing use of nanoplates in biosensing [10] and surface-enhanced Raman scattering detection [11]. 

**Figure 1:**
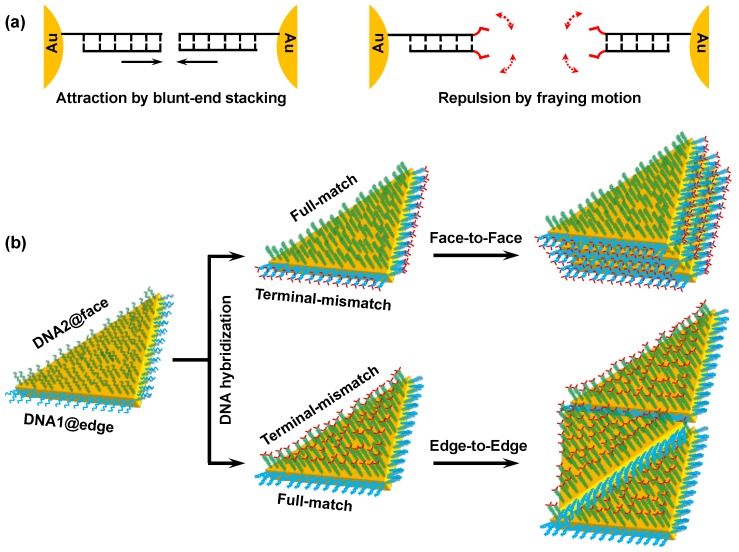
Directed self-assembly of gold nanotriangles (AuNTs) based on interactions between terminal single base pairs of regioselectively grafted DNAs. (**a**) Schematic illustration for attraction between fully matched dsDNAs (**left**) and repulsion between terminally mismatched dsDNAs (**right**), all of which were attached to the gold nanoparticle (AuNP) surface. (**b**) Schematic illustration of the directed self-assembly of AuNTs that were regioselectively modified with fully matched and terminally mismatched dsDNAs.

A self-assembling approach is straightforward and efficient in developing the collective properties that are usually difficult to achieve from individual nanoplates. In previous studies, we discovered unique colloidal behaviors of DNA-modified AuNPs [12]. The AuNPs—functionalized with fully matched double-stranded DNA (dsDNA)—spontaneously underwent rapid aggregation at high ionic strength in a non-crosslinking manner. In sharp contrast, dsDNA-modified AuNPs continued to disperse when dsDNA had a terminal single-base mismatch. The results of colloidal probe atomic force microscopy revealed that the aggregation of the fully matched dsDNA-modified AuNPs was driven by multiple blunt-end stacking of the surface-bound dsDNA, while the high dispersity was attributable to entropic repulsion caused by the fraying motion of terminal mismatched nucleobases (Figure 1a) [13]. The non-crosslinking aggregation behaviors were considered general colloidal phenomena because they were observed for various particles with different morphologies and compositions [14,15].

In addition to applications in sensors and gene diagnostics [16,17], non-crosslinking aggregation showed its potential for the ordered assembly of gold nanorods (AuNRs) by controlling surface modification with DNA [18]. We demonstrated that the AuNRs, regioselectively modified with dsDNA, formed orientation-controlled assemblies in a non-crosslinking fashion. When the dsDNA on the side region of AuNR was fully matched and the dsDNA on the ends was terminally mismatched, the resultant dsDNA-modified AuNRs formed side-by-side assemblies. On the other hand, end-to-end assemblies emerged from the same AuNRs with fully matched dsDNA on the ends and terminally mismatched dsDNA on the side region. The anisotropic reactivity of AuNR surfaces—that enabled the regioselective surface modification—was considered essential for generating these highly directed assemblies.

In the present study, we attempt to achieve orientation-controlled assemblies of triangular gold nanoplates by means of regioselective DNA modification (Figure 1b). Specifically, three edges of gold nanotriangle (AuNT) were modified with one oligonucleotide (DNA1), while two flat faces were covered by a different oligonucleotide (DNA2). Upon formation of the fully matched DNA duplex on the top/bottom faces, along with the formation of terminally mismatched DNA duplex on the edges, the resultant dsDNA-modified AuNTs are expected to preferentially self-assemble in a face-to-face manner. On the other hand, the fully matched DNA duplex formation on the edges and the terminally mismatched DNA duplex formation on the top/bottom faces are expected to allow for selective formation of edge-to-edge assemblies. As the present method is capable of directing AuNT assembly, this suggests it could be generally utilized in the construction of ordered nanostructures.

## 2. Experimental

### 2.1. Chemicals and Apparatus

Nonmodified single-stranded (ss) DNAs were synthesized by Eurofins Genomics and GENEWIZ. 5′- or 3′-mercaptohexyl ssDNAs were obtained from Tsukuba Oligo Service. Molecular beacons (MB1 and MB2) were purchased from Shanghai Invitrogen. All base sequences are provided in Table S1. Gold nanospheres (AuNSs) were obtained from Sigma-Aldrich. Other chemicals were purchased from Wako Pure Chemical Industries, and used without further purification. Fluorescence intensity was measured on a Varioskan Flash Multimode Reader (Thermo Fisher Scientific, Waltham, UK) with an excitation wavelength set at 490 nm and an emission recorded at 520 nm. Transmission electron microscopy (TEM) images were collected on an HT7700 TEM (Hitachi, Tokyo, Japan) or a JEM 1230 TEM (JEOL, Akishima, Japan).

### 2.2. Synthesis of AuNTs

AuNTs were synthesized using a seedless growth method reported elsewhere [8]. Initially, 1.6 mL of cetyltrimethylammonium chloride (CTAC, 0.1 M) and 75 μL of KI (0.01 M) were added to 8 mL of H_2_O. Next, 80 μL of HAuCl_4_ (25 mM) and 20 μL of NaOH (0.1 M) were successively introduced to exhibit a light-yellow color. Then, 80 μL of ascorbic acid (0.064 M) was mixed with the above solutions by shaking, which resulted in a colorless mixture. Finally, 10 μL of NaOH (0.1 M) was injected, and the mixture quickly shaken. After the completion of growth, the thus-obtained AuNTs were purified by depletion flocculation [19]. The AuNT colloid was subjected to two centrifugation/washing cycles to remove excess free CTAC molecules.

### 2.3. Regioselective Modification of AuNTs with ssDNA

Prior to modification, each mercaptohexyl ssDNA was reduced by tris(2-carboxyethyl)phosphine to cleave S–S bonds in the sample as received, and was then purified by ethanol precipitation. For DNA modification, 1 μL of 5′-mercaptohexyl ssDNA (DNA1, 1 mM) was first mixed with the AuNTs (500 μL, 1 nM) at a ratio of 2000:1 by vigorous stirring, followed by incubation for 6 h. Next, 3′-mercaptohexyl ssDNA (DNA2) was introduced to the solution at a ratio of 15,000:1 to the AuNTs, followed by overnight incubation. Then, phosphate buffer (PB, pH 7.4) and sodium dodecyl sulfate were added to give final concentrations of 10 mM and 0.01%, respectively. To enhance the DNA grafting density, the concentration of NaCl in the mixture was gradually increased at intervals of 50 mM, and eventually brought to 300 mM over 6 h (hereafter referred to as the salt-aging step). After overnight incubation, the AuNTs were centrifuged twice and finally dispersed in 5 mM PB (pH 7.4) containing 20 mM NaCl. The modified AuNTs were stored at 4 °C and used within two weeks to preserve its DNA modification regioselectivity.

### 2.4. Characterization of ssDNA-Modified AuNTs

AuNSs with diameters of 15 and 5 nm were respectively functionalized with DNA1-label and DNA2-label mercaptohexyl ssDNAs (Table S1) [12]. To estimate the DNA modification selectivity for AuNT, the ssDNA-modified AuNSs were hybridized to the ssDNA-modified AuNTs at a predetermined ratio. The 15 nm AuNSs were mixed with AuNT at a ratio of 10:1, while the 5 nm AuNSs were added at a ratio of 12:1. Both procedures were followed by overnight incubation in 20 mM PB (pH 7.4) containing 100 mM NaCl at room temperature. TEM analysis was conducted to observe the AuNT–AuNP heteroassemblies. Statistical analysis was performed to evaluate the regioselectivity of DNA modification.

To determine the total number of surface-grafted DNA1 and DNA2, the AuNTs were subjected to centrifugation after the salt-aging step in the DNA modification procedure. Next, 50 µL of the supernatant was incubated with 100 µL of MB1 (Table S1) at 65 °C before annealing to room temperature, in which 13 mM PB (pH 7.4) containing 130 mM NaCl was included. The restored fluorescence intensity was compared to that obtained by incubation of MB1 with DNA1 at the initial concentration used for AuNT modification. This procedure allowed for calculation of the amount of DNA1 attached to the AuNT surface. Similarly, the number of DNA2 was estimated by measuring the fluorescence intensity of the supernatant mixed with MB2 (Table S1).

### 2.5. Formation of Directed dsDNA-Modified AuNT Assemblies

To obtain the non-crosslinked assemblies of the dsDNA-modified AuNTs, 2 μL of the ssDNA-modified AuNTs (2.7 nM), 2 μL of cDNA1 or cDNA1′ (2 μM), and 2 μL of cDNA2 or cDNA2´ (2 μM) were mixed prior to incubation at room temperature for 10 min. The final concentrations of the AuNT and NaCl were 0.9 nM and 6.7 mM, respectively. Then, 3 μL of the resultant dsDNA-modified AuNTs were dropped on an elastic carbon-coated copper grid (ELS-C10, Okenshoji, Tokyo, Japan) which had been hydrophilized using UV/ozone treatment. The droplet was left for 2 min without disturbance, followed by removal of the solution with a piece of filter paper. TEM measurements were performed under an accelerating voltage of 80 kV.

## 3. Results and Discussion

### 3.1. Synthesis and Characterization of ssDNA-Modified AuNTs

**Figure 2:**
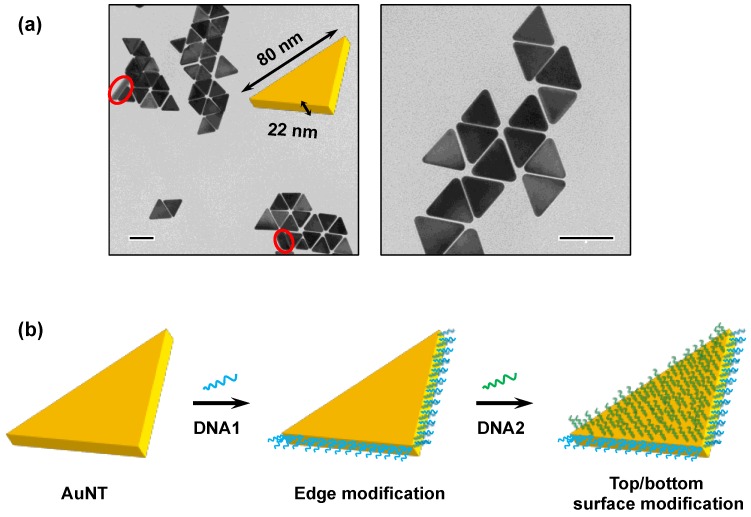
Characterization and surface modification of the AuNTs. (**a**) Typical TEM images of the seedless grown AuNTs. Red circles highlight the AuNTs standing vertically on the TEM microgrid. Scale bars are 100 nm. (**b**) Regioselective DNA modification of AuNT by successive addition of DNA1 and DNA2.

The AuNTs used in this study were grown via a seedless growth pathway and were surface-capped by CTAC [8]. As shown in Figure 2a, TEM analysis revealed that the thus-obtained AuNTs were of uniform size, having an edge length of approximately 80 nm. By analyzing the AuNTs that stood vertically on the surface of the TEM microgrid, the thickness of the AuNTs was estimated to be approximately 22 nm, on average. Due to the convex surfaces and the less stable crystal facets, the edges exhibited a higher reactivity than the top/bottom flat surfaces [20]. As a consequence, after the addition of DNA1, the CTAC capping on the three edges was preferentially replaced by DNA1 strands through Au–S bond formation (Figure 2b). Subsequently, excess DNA2 strands replaced the CTAC molecules on the top/bottom flat surfaces.

**Figure 3:**
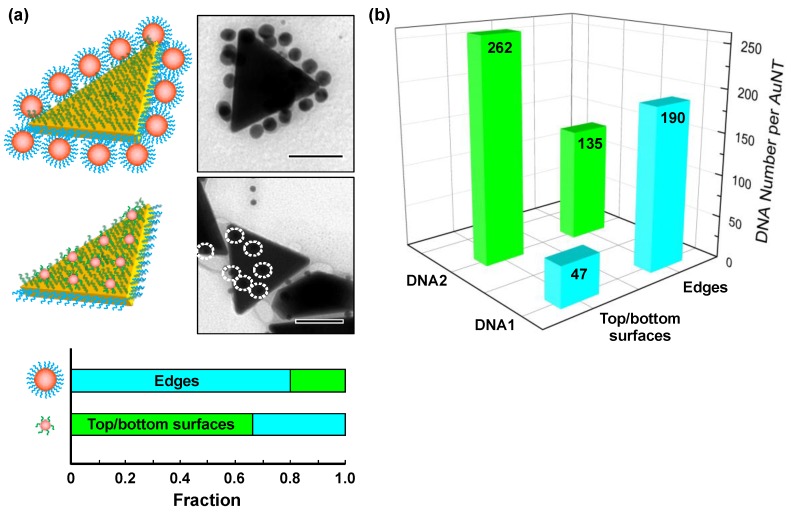
Characterization of the ssDNA-modified AuNTs. (**a**) Typical TEM images for the edge-attachment of 15 nm AuNSs (top) and for the flat surface-attachment of 5 nm AuNSs (bottom) to the AuNT. Scale bars are 50 nm. Degrees of regioselectivity in DNA modification on the AuNT surfaces are also shown. (**b**) The numbers of DNA1 and DNA2 grafted onto the edges and the top/bottom surfaces of a single AuNT.

In order to estimate the regioselectivity of the ssDNA modification, DNA-crosslinked heteroassemblies of AuNT and AuNS were prepared for statistical analysis. For this purpose, AuNSs with diameters of 15 and 5 nm were functionalized with DNA1-label and DNA2-label, respectively (Table S1). Upon crosslinking of the AuNTs with the 15 nm AuNSs, we observed that the three edges of the AuNTs were selectively covered with the 15 nm AuNSs (Figure 3a and Figure S1a). A survey of over 300 AuNSs (15 nm) by TEM suggested that the modification selectivity of DNA1 onto the edge regions of the AuNTs was 80%, indicative of the fairly high reactivity of edge regions. Similarly, about 160 binding events of the 5 nm AuNSs to the AuNTs were analyzed, resulting in an obtained modification selectivity of 66% (Figure 3a and Figure S1b).

Subsequently, we determined the total numbers of DNA1 and DNA2 attached to the whole AuNT surfaces to be 237 ± 15 and 397 ± 19, respectively, by using a molecular beacon-based method [18]. The total DNA grafting density on the AuNT surfaces was calculated to be 0.059 strands/nm^2^, which was similar to the value reported in a previous study [19]. Taking these results together, the number of DNA1 on the edges and DNA2 on the top/bottom surfaces were determined to be 190 and 262, respectively (Figure 3b). Overall, the DNA numbers on the edges and the top/bottom surfaces were estimated to be 325 and 309, respectively. The DNA grafting density on the top/bottom surface (0.056 strands/nm^2^) was slightly lower than that on the edge (0.062 strands/nm^2^). This was probably attributable to higher steric and electrostatic repulsion between adjacent DNA strands on the flat surfaces.

### 3.2. Directed Self-Assembly of dsDNA-Modified AuNTs

Next, we attempted to construct directed assemblies of the DNA-modified AuNTs by controlling the terminal base pairing/unpairing of DNA1 and DNA2. The duplex formation of DNA1 and DNA2 with their fully matched or terminally mismatched sequences enabled us to control the outermost base pairing of the dsDNAs on the edges and the top/bottom surfaces of the AuNTs. Given the small number ratio of DNA1 (15%) on the top/bottom surfaces, the terminal base pairing/unpairing of DNA2 should dominate the interfacial property of these regions. Moreover, the undesired influence of DNA2 base pairing/unpairing on the edges should be exercised marginally, even though the DNA2 strands represented about 40% of the total number of DNA grafted onto the edges (Figure 3b). This is because the DNA1 strand (25 nt) was sufficiently longer than the DNA2 strand (16 nt). Our previous study demonstrated that the status of the outermost base pairing/unpairing of the longer surface-grafted dsDNA governed the colloidal behaviors of the dsDNA-modified AuNRs [18].

**Figure 4:**
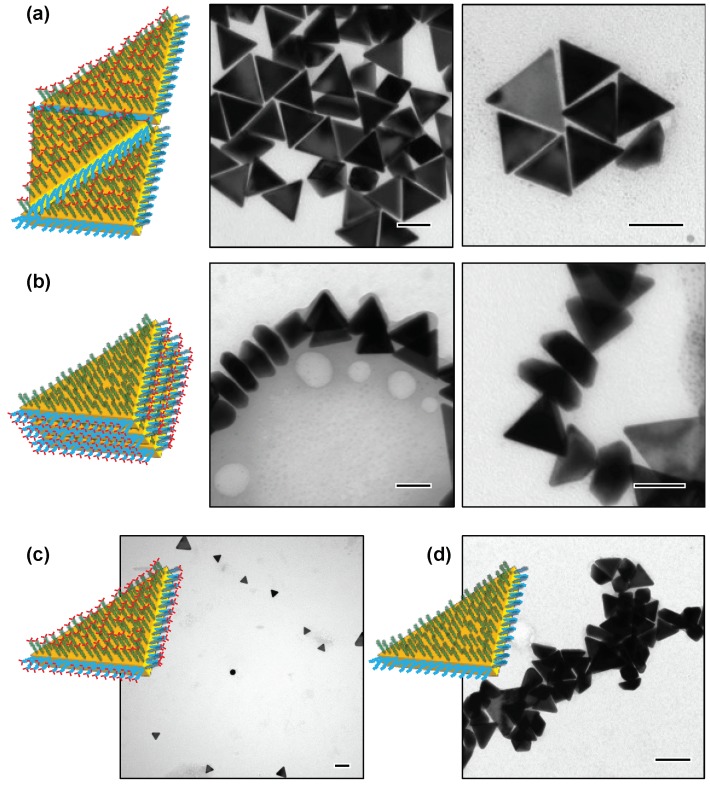
Assembling behaviors of the AuNTs controlled by terminal base pairing of surface-grafted DNA. Representative TEM images for (**a**) the edge-to-edge assembly of the AuNTs modified with fully matched dsDNA on the edges and terminally mismatched dsDNA on the flat surfaces, (**b**) the face-to-face assembly of the AuNTs modified with terminally mismatched dsDNA on the edges and fully matched dsDNA on the flat surfaces, (**c**) stable dispersion of the AuNTs modified with terminally mismatched dsDNA on all surfaces, and (**d**) undirected assembly of the AuNTs modified with fully matched dsDNA on all surfaces. Scale bars are 100 nm.

When DNA1 and DNA2 were hybridized with cDNA1 and cDNA2´ (Table S1) to form fully matched and terminally mismatched duplexes, respectively, edge-to-edge assemblies of the AuNTs were produced in a non-crosslinking fashion (Figure 4a and Figure S2). On the other hand, when the AuNTs were functionalized with the terminally mismatched dsDNAs on the edges and the fully matched dsDNAs on the top/bottom flat surfaces, they spontaneously formed face-to-face assemblies (Figure 4b and Figure S3). TEM imaging revealed that the dsDNA-modified AuNTs not only stacked in parallel, but also stood vertically on the surface of the TEM microgrid to form the face-to-face assemblies. In control experiments, the AuNTs modified with terminally mismatched dsDNAs on both the edges and the flat surfaces showed high colloidal stability, even in the presence of 300 mM NaCl (Figure 4c). As expected, the AuNTs modified with fully matched dsDNA on both the edges and flat surfaces were spontaneously aggregated in an undirected manner (Figure 4d). These results underscored the important role of the terminal base pairing status of DNA1 on the edges and DNA2 on the top/bottom surfaces for generating directed AuNT assembly. As with AuNRs, AuNTs were also able to produce highly directed assemblies by making use of the non-crosslinking aggregation behaviors.

It is worth pointing out that the unmodified AuNTs used in the present study preferentially formed edge-to-edge assemblies, probably due to the moderate aspect ratio (3.6) between the edge length and the thickness (Figure 2a). Therefore, the selective formation of face-to-face assemblies by the present method verified that strong attractive forces took place between the surface-bound fully matched dsDNAs by virtue of multiple blunt-end stacking interaction. In line with this consideration, the grafting density of DNA2 enormously affected the face-to-face assembling behavior of the AuNTs. Decreasing the amounts of DNA2 from 15,000 to 6000 equivalents in the preparation procedure resulted in a lower yield of the face-to-face assembly without a remarkable change in yield of the edge-to-edge assembly, albeit with a more significant decrease in the amount of DNA1 from 2000 to 500 equivalents (Figure S4). The result strongly suggested that the degree of top/bottom surface modification with fully matched dsDNA played a key role in the construction of directed face-to-face AuNT assemblies.

## 4. Conclusions

In summary, we demonstrated that AuNTs could be modified with ssDNA in a regioselective fashion, thereby facilitating their controlled non-crosslinking assembly. The regioselective DNA modification was accomplished by means of a simple two-step procedure, which exploited the uneven surface reactivity of AuNT. The results of TEM imaging revealed that the dsDNA-modified AuNTs formed face-to-face assemblies when the dsDNA on the top/bottom surfaces was fully matched and the dsDNA on the edges was terminally mismatched. By contrast, the edge-to-edge assemblies emerged with fully matched dsDNA on the edges and with terminally mismatched dsDNA on the top/bottom surfaces. The assembling process was probably driven by blunt-end stacking interaction on an intentional region of the AuNTs, along with repulsion caused by the fraying motion of terminal mismatched nucleobases in the other region. A major advantage of the present approach is that assemblies of differing orientation can be obtained by using strictly identical building blocks. Indeed, this has been achieved with limited success, and has been helpful in making strict comparisons of physicochemical properties between different directed assemblies. The present study suggests that the directed non-crosslinking assembly can be extended to other nanoparticles that exhibit uneven surface chemical properties.

## Supplementary Materials

The following are available online at https://www.mdpi.com/2079-4991/9/4/581/s1. Table S1: Base sequences of the surface-grafted DNA, complementary DNA, and molecular beacon. Figure S1: Additional TEM images for the heteroassemblies of AuNT and AuNSs. Figure S2: Additional TEM images for the edge-to-edge assemblies of the dsDNA-modified AuNTs. Figure S3: Additional TEM images for the face-to-face assemblies of the dsDNA-modified AuNTs. Figure S4: Typical TEM images for the assemblies of dsDNA-modified AuNTs with reduced DNA-grafting densities.
