# Supplementary material for: Regioselective DNA Modification and Directed Self-Assembly of Triangular Gold Nanoplates
Source: Nanomaterials (Basel). 2019 Apr 9;9(4):581. doi: 10.3390/nano9040581 (PMC6523274; doi:10.3390/nano9040581)
Supplement: Supplementary file 1 [file nanomaterials-09-00581-s001.pdf]

Supplementary Materials for

Regioselective DNA-Modification and Directed Self-Assembly of Triangular Gold Nanoplates

Guoqing Wang*, Yao Zhang, Xingguo Liang, Tohru Takarada*, and Mizuo Maeda

Table S1. Base sequences of the surface-grafted DNA, the complementary DNA, and the molecular beacon.

Length	Code	Sequence Information [a]
25 nt	DNA1	3′-CCTCGACCACCGCATTATTCCTCAT-5′-**SH**
25 nt	cDNA1	5′-GGAGCTGGTGGCGTAATAAGGAGTA-5′
25 nt	cDNA1′	5′-CGAGCTGGTGGCGTAATAAGG AGTA-5′
16 nt	DNA2	5′-TCGCAACAATAACTGA-3′-**SH**
16 nt	cDNA2	3′-AGCGTTGTTATTGACT-5′
16 nt	cDNA2′	3′-TGCGTTGTTATTGACT-5′
25 nt	DNA1-label	3′-AATGCGGTGGTCGAGGTTTTTTTTT-5′-**SH**
16 nt	DNA2-label	5′-ATTGTTGCGATTTTTT-3′-**SH**
39 nt	MB1	**FAM**-5′-TTGATCTGGAGCTGGTGGCGTAATAAGGAGTAAGATCAA-3′-**BHQ-1**
24 nt	MB2	**FAM**-5′-CCTCTCAGTTATTGTTGCGAGAGG-3′-**BHQ-1**

[a] SH, FAM, and BHQ-1 denote the mercaptohexyl group, 6-carboxyfluorescein, and Black Hole Quencher 1, respectively. The mismatched terminal bases in cDNA1′ and cDNA2′are highlighted in red.

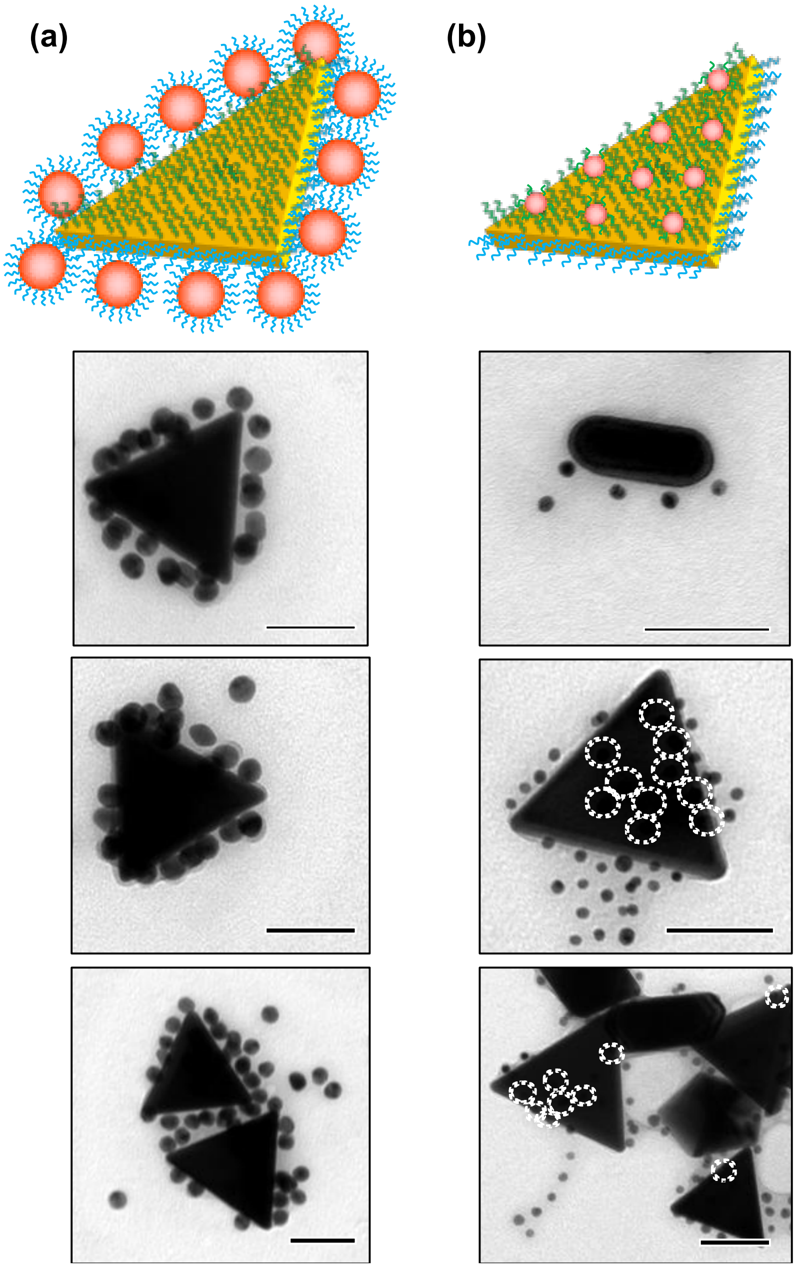

Figure S1. Additional TEM images for the heteroassemblies of the AuNT and the AuNSs with diameters of (a) 15 nm and (b) 5 nm, respectively. Scale bars are 50 nm.

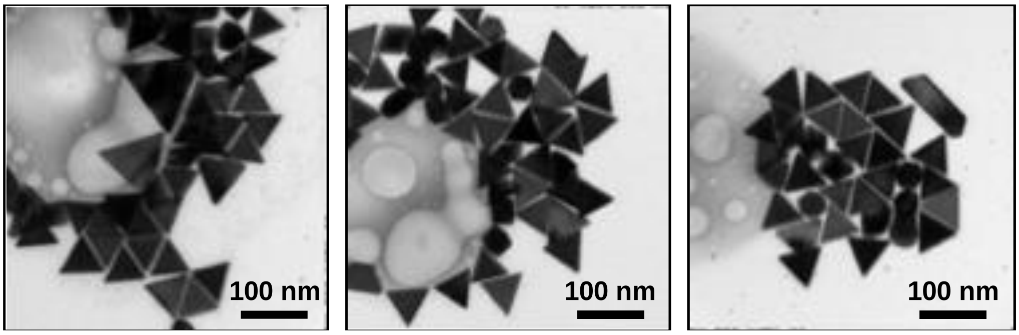

Figure S2. Additional TEM images for the edge-to-edge assemblies of the dsDNA-modified AuNTs.

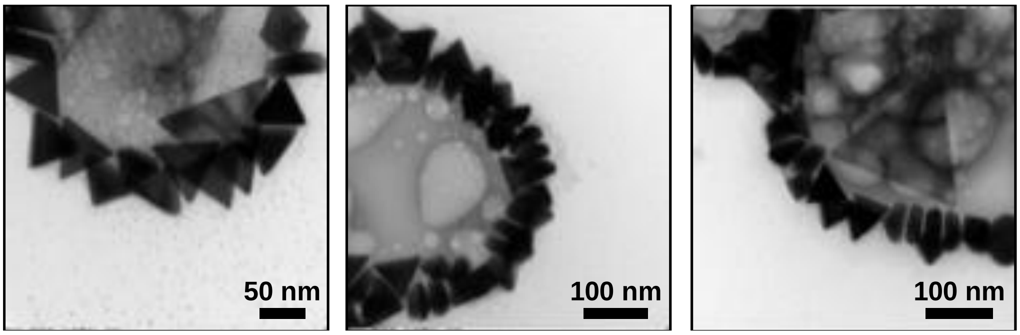

Figure S3. Additional TEM images for the face-to-face assemblies of the dsDNA-modified AuNTs.

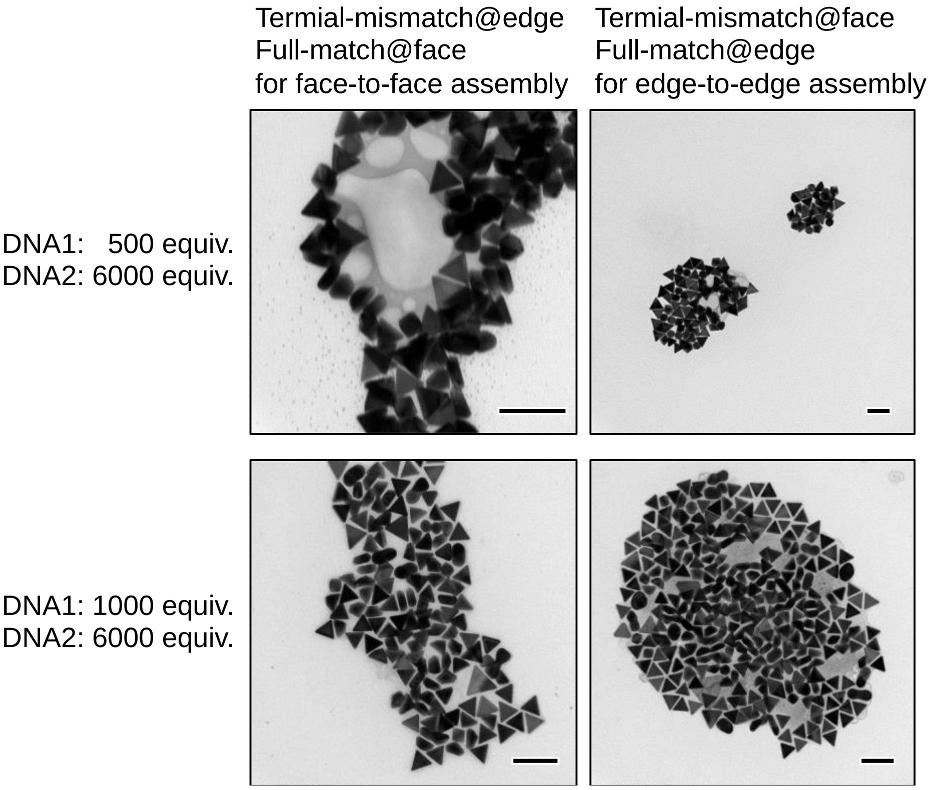

Figure S4. Typical TEM images of the assemblies of the dsDNA-modified AuNTs with reduced DNA-grafting densities (original: 2000 equiv. of DNA1 and 15000 equiv. of DNA2). Scale bars are 100 nm.
